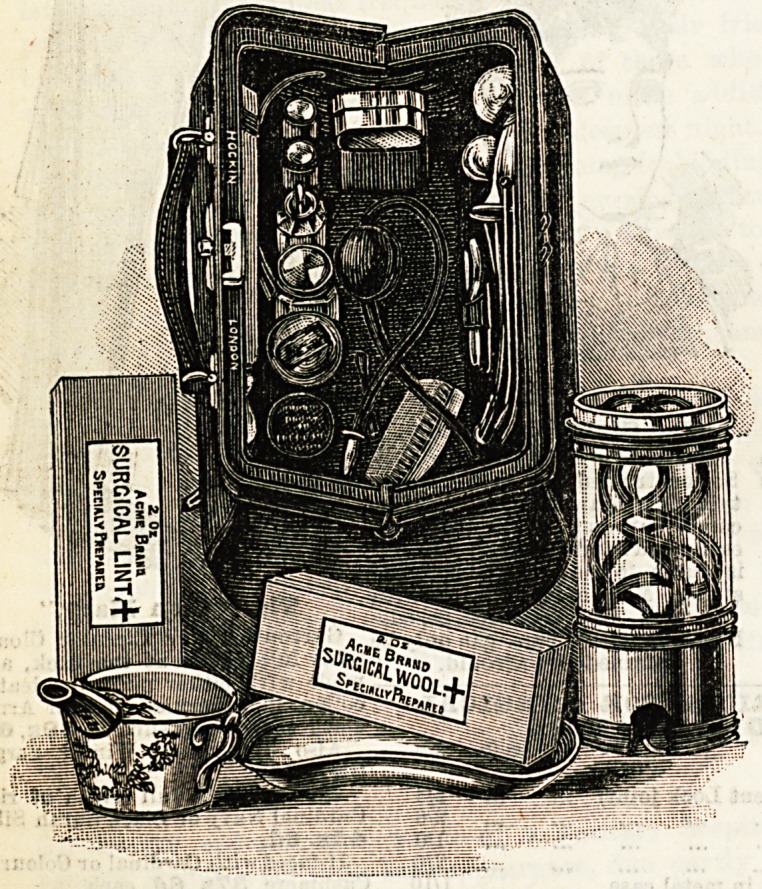# The Hospital Nursing Supplement

**Published:** 1894-11-24

**Authors:** 


### "The Hospital" Nursing Mirror.

Being the Extra Nursing Supplement of "The Hospital" Newspaper.

[Contributions for this Supplement should be addressed to the Editor, The Hospital, 428, Strand, London, W.C., and should have the word "Nursing" plainly written in left-hand top corner of the envelope.]

### News from the Nursing World.

### OUR PRINCESS IN RUSSIA.

Each stage of the long Russian journey taken by the Princess of Wales has been eagerly followed by many Hospital readers. That her presence and that of the Prince of Wales and the Duke of York have been of infinite comfort to the Czar and his Royal mother is sufficiently obvious; although the fatigue of days full of such sad excitement must have tried the endurance of all. A special funeral service held at the Anglican Church, St. Petersburg, on 18th inst., was attended by their Royal Highnesses the Prince and Princess of Wales, the Duke of York, and the Duke of Coburg; and it was afterwards decided that a subscription should be opened to endow two beds in a hospital which bears the name of the late Czar.

### LEWISHAM UNION INFIRMARY.

Things have speedily come to a crisis at this Infirmary. At a meeting of the Guardians, on the 15th inst., the Infirmary Committee reported that for some time past there had been a certain amount of friction between the Medical Superintendent and the Matron on matters of administration; that as the result of an investigation into certain matters complained of, they regretted to have to record that the Matron had been insubordinate, and assumed an authority upon matters of discipline in opposition to the views and expressed wishes of the Medical Superintendent. After careful consideration the committee expressed the opinion that the Matron's action "had been subversive to the authority of the Medical Superintendent, prejudicial to the health of a patient in one instance, and tending to make discipline most difficult." They suggested the advisability of her resigning her appointment, unless she could distinctly recognise that the authority of the Medical Superintendent is paramount in all respects within the limits of the order of the Local Government Board, and act accordingly. The Matron, Miss Pattison, who has a high reputation for ability and energy, has appealed to the Local Government Board. She was called before the Guardians and asked by the chairman if she would recognise the authority of the Medical Superintendent as paramount, and act under his authority. We have not space to give the encounter which took place between the chairman and the Matron, but the latter's main answer will sufficiently indicate the position she takes up. The Matron said: "I am asked whether I will accept and carry out the Medical Superintendent's instructions, if they are reasonable or unreasonable, whether right or wrong. Certainly not." She then stated that she did not trust the Medical Superintendent. The Guardians suspended her in consequence by eleven votes to three; and Miss Mills, one of the assistant matrons, was appointed to take the duties of Matron *pro tem.* It is impossible to go into the merits of the case until the evidence has been heard at the inquiry. It is much to be regretted, however, that in so short a time matters should have been brought to such a crisis, but all must agree that where two people ride on a horse one must ride first.

### OUR CHRISTMAS COMPETITION.

The time is very short until Christmas arrives, and whether it finds us well or ill supplied with gifts for the sick in hospital wards depends to a great extent on the goodwill of our readers. We venture to anticipate a larger supply of parcels than has ever before been received at the office. The prizes offered are: 20s. for the most serviceable dressing gown; 10s. for the best flannel shirt; 7s. 6d. for the best flannel petticoat; 7s. 6d. for the best over petticoat; 7s. 6d. for the best bed jacket; 5s. for the best knitted pair of men's socks; and 2s. 6d. for the second best pair. We hope those who do not care to compete will contribute useful garments of all descriptions, and send them also to Nursing, care of Editor, 428, Strand, by December 18th.

### PROGRESS AT READING.

A good example is being set by the Reading Guardians, who have agreed to raise their head nurse's salary and to augment the staff by the addition of two probationers. The latter will be paid £10 during the first year, £15 the second, and £20 the third, and they will be supplied with uniform. A Nursing Committee, consisting of Mrs. Bailey, Mr. G. R. Smith, and Mr. D. J. Cooke (chairman of the Board), has been appointed, and Dr. Guilding (the medical officer) will arrange for the probationers to attend nursing lectures given at the hospital.

### NURSING AS AN INDUSTRY.

Under the title of "Nursing," an interesting lecture was given at the South Place Institute on November 18th by Miss Honnor Morten. It formed one of a course on "Industries," and the speaker gave a comprehensive history of the progress of nursing from early until present times. The work, hours, and earnings of hospital and private nurses were graphically described, and registration for midwives and training for asylum attendants were advocated. The audience evinced great interest in the whole lecture, and loudly applauded Miss Morten's description of nursing in the Crimea. Each time Miss Nightingale's name was mentioned it was received with loud applause.

### THE BLACKBURN INFIRMARY NEEDLEWORK GUILD.

This needlework guild, of which Mrs. Henry Harrison is the indefatigable hon. secretary, is a most valuable adjunct to Blackburn Infirmary. By means of it a warm garment is supplied to every in-patient on Christmas Day, and it also enables the matron, Miss Hale, to see that each rheumatic and phthisical patient is provided with a change of woollen underclothing on leaving the hospital. The guild sent in 570 finished articles last year, and after supplying the special cases referred to, the remainder of the things are reserved as hospital property, and loaned to necessitous patients during their stay in the wards. At the annual meeting of the needlework guild the matron furnishes a list of the garments most urgently needed. She remarks truly, "Blackburn Infirmary is indeed favoured, and I wish that every hospital had its own needlework guild." But so far very few institutions have even a fraction of such help, and it remains with our readers to ensure to every inmate of our hospitals on Christmas Day a suitable gift, such as those invited by our needlework competition.

### A FRESH VERSION.

The Cardiff Guardians are satisfied, according to the local press, that the sick poor are properly looked after in their workhouse. They evidently consider that the jury did not understand the facts, or the nurse, at the inquest referred to under the heading, "Where was the doctor?" in The Hospital of Nov. 10. In the rider to the verdict, attention was called to insufficient attendance in the infirmary at night (one nurse to ninety-four patients, besides "the poison case"). From that report it appeared that the dying man had neither medical attendance nor nursing, although the police-constable swore to his critical and suffering condition. Still the guardians "are perfectly satisfied"; the nurse has explained that the jury misunderstood her, she did not complain of being overworked, and "she saw the patient about every hour." It is somewhat difficult to reconcile the recent with the former account of the case, but doubtless the ratepayers will need convincing as well as the guardians that the patient had adequate care and attention before he expired from the effects of the arsenic he had taken.

### AN EMERGENCY WELL MET.

It is reported that eleven trained nurses have been engaged for the service of the poorer inhabitants of the town of Newport (I. of W.), during the present typhoid epidemic. The promptness with which nine were installed, to work immediately under the doctors, is mainly due to the energy and liberality of a lady at Shanklin, who is said to have given a donation and promised another towards defraying the expense thus involved. A public subscription has been set on foot and a relief fund organised, sums also being voted to each district to provide sick-room necessaries.

### QUEEN'S NURSES IN SCOTLAND.

Good are the latest tidings received from the Hamilton branch of the Queen's Jubilee Institute, for a donation of £1,000 has been paid towards its endowment. Mrs. Ford Simpson and Mrs. William E. Simpson have made this handsome gift "as a memorial of their families' former connection with the town." It is probable that the funds which have been raised to perpetuate the memory of the late Mr. J. C. Forrest may be dedicated to endowing the Nurses' Home. Nurses MacMaster and Philp paid 5,385 visits during the year to 236 patients.

### WANTED—A LITTLE HELP.

The Troon District Nursing Association is in want of help, for which it certainly ought not to have long to wait. Many visitors who go in the summer to this pleasant spot will no doubt gladly contribute some of the warm garments so urgently needed by the sick folks at present. All contributions sent for the use of the patients will be gratefully welcomed by the Association, which, although only established last spring, is already much valued in the district.

### IRISH ENTERTAINMENTS.

The Governors of the Monaghan County Infirmary gave a ball in the Town Hall, Monaghan, last week, at which over one hundred guests were present, and the committee are to be congratulated on the success of an evening which materially aided the funds of a most useful institution. The annual conversazione of the Dublin Naturalists' Field Club was held on Tuesday evening at the Royal Irish Academy, Dawson Street, the chair being taken by Mr. G. H. Carpenter, B.Sc., the president. A novel feature was introduced in a miniature garden, "The Folk-lore of Fungi," designed by lady members. Dr. McWeeny gave an interesting lecture on the bacilli of various diseases, illustrating each group by means of lantern slides. An endeavour to supplement the funds of the "Cruelty to Children" Association will be made on December 4th by a doll-dressing competition, to be held at the Molesworth Street Shelter. All competitions ready by November 20th will be judged by Mrs. Power Lalor and Miss Bereford. One class of dolls, eighteen inches high, will be dressed as "hospital nurses."

### SHORT ITEMS.

Miss Iza Coghlan, M.B., of Sydney, was recently made medical referee for the Australian Mutual Provident Society.—The Duchess of Sutherland has been re-elected president of the Longton Sick Nursing Association, which had a good record of work to submit to the subscribers at the recent annual meeting.—It is under discussion to engage a trained nurse for Kilmallock from the Irish Branch of Queen's Jubilee Institute.—At the Conference of Women held in Dundee, Miss Wood, of Glasgow, spoke, in the Scottish division of the Y.W.C.A., of the "Nurses' Union," which she described as a Nurses' Christian Association, started three years ago by Mrs. Oatts.—St. Lawrence's Catholic Home for Nurses for the Sick Poor at Dublin has moved into new premises; 34, Rutland Square West, having been placed by Lady O'Hagan at the service of the committee.—Mr. Bannister has given a bath-chair to the Grimsby and District Nursing Association for the patients' use.—Miss Honnor Morten has resigned the post of hon. secretary of the Nurses' Co-operation, 18, New Cavendish Street.—The new dispensary and wards of the Hospital for Women at Liverpool were formally opened on 16th inst. by the Earl and Countess of Derby.—An entertainment in aid of the Nursing Association at Atherstone was recently held at the Corn Exchange in that town, when an interesting programme was carried out before a large and enthusiastic gathering.—The Aberdeen District Nursing Society has made rapid progress, and the superintendent, Miss Armstrong, has five nurses now working with her.—The Princess Mary Village Homes, established for the training of the female children of criminals, require subscriptions for extra structural repairs.—Miss Hickman, Lady Superintendent of the Kidderminster Nursing Institute, explains that the nurse remarked on at recent board meetings as not trained, although her services were charged for as such to the institution, is not on her staff. She was merely engaged, at the urgent request of the medical officer of the workhouse, for one week in an emergency, and it does not appear that she was inefficient. The fee of a guinea which was due to her, therefore in no way benefited the nursing institution; and, as the chairman, Mr. Brinton, remarked, it is only justice to Miss Hickman to state publicly that it is not her custom to send out untrained nurses.—Miss Isabel Dodgson has given three lectures on sick nursing at Kidlington, which have been well attended and enthusiastically received; illustrations by means of a magic lantern forms a special feature in Miss Dodgson's programme.

### The American Woman at Home.

By Our Own Correspondent.

(*Continued from page* xlv.)

### II.—HER FOOD.

I have said that part, at least, of the difficulties of the servant question in America arise from the fact that the servants are too few in proportion to the work. An overworked servant is never a good one. Tyranny cannot breed loyalty. One of the things for which we have to thank our aristocracy—a thing which one appreciates most in a republican country—is that they have set the fashion of consideration towards dependants, patronising consideration if you will, but still something that brings about a personal relation between master and man, mistress and maid. The fashion thus set filters through all ranks, except perhaps the lowest servant-keeping class, where, as a rule, the service is bad, insolence the rule, and theft and drunkenness by no means uncommon. In estimating the work of an American cook, for example, it may be worth while to analyse the ordinary menu of an American family, studying its effect upon the servant's muscles, and the mistress's digestion and temper.

The average breakfast, in a modest house, where only one maid is kept, or perhaps none, is something like this:—Fruit, porridge of oatmeal, hominy, or some other cereal, beef-steak, fried potatoes, hot cakes of some kind with preserve or maple syrup, coffee, and iced water—that first essential of American liking. This is a fairly solid meal for a man who has hard manual work to do; but think of it as beginning the day for a delicate woman whose heaviest labour will be an interview with her dressmaker! It is no wonder that by the plate of the lady of the house you will often see, besides these dainties, a medicine bottle and a spoon. Tonics, digestives, purgatives, are almost as essential a part of American living as the potato, which, as in the traditional fare of Ireland, appears at every meal. Add to the mere quantity of food here set down the fact that the steak is often tough and overdone, and that steel knives being things unknown, it must be torn in fragments with a silver or plated knife, and it will be seen that enough work is left for mastication and digestion to do.

For dinner at noon there is soup, meat, with an endless variety of vegetables, pie served with cheese, salad, and very probably ice-cream. The soup is usually good—transparent consommé, or white soup made largely of milk, oyster or clam soup, or the very agreeable and nourishing black bean soup. The meat is distinctly inferior to British; it is often tough and stringy, and even when tender it lacks flavour. For vegetables we have potatoes once more, and at least two, often three or four, vegetables beside—for example, asparagus, peas, beetroot, and cold slaw (a sort of salad made from finely-chopped cabbage). These are all served in separate dishes surrounding the central dinner-plate, and you go dabbing for this or that at your caprice. This fashion must, I think, have been the originator of the American method of eating, which demands that the fork shall be held in the right hand. Busy man cuts, or rather tears, his food into convenient pieces with the thing miscalled a knife, then takes up his fork once for all, and bolts his food with marvellous celerity. Daintier woman drags off one fragment of meat at a time, then transfers the fork to her right hand to carry the meat to her mouth and take whatever complement of vegetables she desires, gives the fork back to the left hand, takes up her knife once more to cut another piece, and so on till an end is reached. Pie deserves a word to itself. It consists of a thin layer of fruit between two solid layers of paste. Pie plays an important part in American economy. It is the universal stop-gap. If childhood develops the fierce and unreasoning hunger to which it is subject between meals, that hunger is appeased, not by bread and butter (which often satisfies it for ever), but by pie. Lunch (and lunch means any irregular meal served between dawn and midnight), lunch means pie. In all the awkward crevices of appetite is pie inserted; and pie continued through a lifetime means dyspepsia.

The action of pie is universal—it is felt in the remotest districts. The backswoodsman and the miner, as well as the citizen, suffer alike from a plethora of pie. But in the cities its successful rivals, especially with womanhood, are ice-cream and candy. The quantity of ice-cream an American woman can consume would make the North Pole shiver; her taste for candy makes one tremble for the future of the sugar-cane. Not content with ending off a dinner with ice-cream, and taking it as a restorative, either *au naturel* or mixed with soda water, at any hour when she finds herself within reach of a place where it is sold, the American woman has the cruel habit of, midway through a feast, offering her guests a thing she calls a punch or sorbet, which is, indeed, no other than a half-frozen water-ice flavoured with rum. This is supposed to renew the appetite, satiated with what has gone before. Perhaps it does so; but at what a price of future penitence. Candy is, of course, a thing to be consumed at any hour of the day. It is no monopoly of youth; in fact it is a whim of the American mother (who consumes pounds of sweetmeats every week herself) to keep her children from them. She would be wise for them if not for herself; but alas! example is stronger than precept, even when it is not better, and till Madame Mère stops her own indulgence, it is to be feared that her children will crave for candy too. Of the horors of "chewing gum," a sticky mass flavoured with fruit, and (it is said to excuse its existence), doctored with pepsin, I cannot speak. That it is largely consumed I am told, but the lack of elegance attending its consumption keeps it in the background. I have never seen it eaten, but I have seen a grandmother devouring pop-corn and pea-nuts with an avidity more resembling six than sixty.

I believe that these eccentric dainties take the place of the wine which the American woman does not drink. She is almost fanatically "temperance" in her attitude. She will fanatically "temperance" in her attitude. She will give her husband tea—green tea—with his dinner, and if he suggests a preference for a glass of beer, will pathetically beseech him not to acquire "the liquor habit," totally forgetting, or knowing nought of the "cock-tails" and other strange "mixed drinks" he has consumed an hour before in the "sample room" (euphemism for bar) of some hotel. In the dining-room of an American hotel you will see a hundred men at dinner, drinking nothing stronger than iced water. A pint of claret or a glass of beer will almost infallibly proclaim the foreigner. But do not think that all these Yankees are abstainers. For my own part I think that drinking between meals leads to the worst forms of alcoholism, but my American dame is an ostrich, who, seeing no intoxicant on her table, believes that her menkind do not touch it.

I have spoken of breakfast and dinner, and supper resembles them much—tea, meat, fried potatoes, and fruit, with bread and various kinds of cake. It will thus seem to the English mind that there is a monotony about American food; but I am assured that Americans complain equally that our food is monotonous. So much depends on habit! But for my own part I don't enjoy potatoes three times a day, nor green tea at any time, and this, unless you specially state your preference for "English breakfast tea" is what is invariably set before you. Of course, Americans who have travelled in Europe often vary very considerably their mode of life, *à l'anglaise* or *à la française*, according to individual taste; but I have here endeavoured to describe the ordinary food of an ordinary American household. Afterwards I will speak of more ceremonious meals.

### Notes from Germany.

An annual sum of M.4,000 (£200) has been granted by Government to the nursing department of the Women's Society in Baden for the supply of trained nurses in epidemics.

The sad death of the young physician, Dr. Oerter, recently occurred in Hamburg, in which city he distinguished himself at the time of the cholera epidemic by his untiring devotion to the sick and suffering. Since then he has made the disease his especial study, and it was while working on the bacteria that he contracted the disease of which he died.

Herr Professor von Liemssen recently drew attention to the want of consideration and tact displayed by visitors to patients in hospital. He would like each to limit a visit to a quarter of an hour; to allow nothing in the way of food unless authorised by the physician or nurse; and for guests to walk and speak quietly. He also suggests that the relatives make known to the sister in charge or the doctor any wish expresssed by the patients, as through the reticence of the latter misunderstandings frequently arise. He wishes the patients to be in no way debarred from seeing their friends, but makes these suggestions on behalf of those who are seriously ill, to whom unnecessary noises mean additional pain, an elevation of temperature, and a sleepless night.

A Bill has just been passed by the Reichstag forbidding the erection of a hospital, asylum, or private nursing institution in any locality in which it might prove a nuisance to the neighbourhood. For instance, should it be proposed to erect an infectious or a Roman Catholic hospital in a locality where either would be a grievance to the neighbourhood, another site must be selected.

Towards the prevention of the further spread of tuberculosis, the Bavarian Ministry prescribes the following measures: All rooms set apart for the reception of tuberculous patients are to be furnished in such a manner that they can be easily scrubbed throughout—walls included. No carpets are permitted. All body and bed linen to be kept apart and washed separately, and be cleansed and disinfected by boiling, steam disinfection, chemical treatment, or exposure to air. Articles of no great value are to be destroyed after contact with the patients, and no clothes are to be given away or sold before being disinfected. Unboiled milk is prohibited. All new institutions for the treatment of tuberculosis are henceforth to be erected with the view of isolating the patients, in rooms which can be thoroughly ventilated and aired daily. Only spitoons made of glass, earthenware, or enamel are permitted to be used by such patients. These precautions ought to be effectual in diminishing this disease.

Herr Oscar Rocholl, of Berlin, has invented several temporary hospitals, which, should they come into general use, will obviate some of the difficulties now attendant on limited nursing accommodation. The smaller building designed by him is on the same principle as an ordinary structure, and can be enlarged by means of lines laid on each side, by which two wings provided with wheels can be drawn out from the main structure when required. This addition could be used as day-rooms for convalescents. The second model is on a much larger scale, and the rooms are arranged in a block, the whole of the ground floor admitting of extension; three sides of the rooms are of glass, set in a strong wooden framework. Any of these rooms may be entirely separated from the main structure and wheeled into the garden, thus being adapted for isolation purposes. All the apartments are on castors or small wheels, which run smoothly on lines laid down for them. A sick pavilion, also constructed by Herr Rocholl, revolves instead of extending, and contains three or four small rooms; it is approached by steps, and might be erected in a garden.

Professor Leyden resumed his lectures on November 9th. The auditorium was crowded with students, who cheered enthusiastically at his reappearance amongst them. In returning thanks for this hearty welcome the professor said that the time spent in the Crimea, in the pursuance of his difficult and serious task, formed a period which would never be obliterated from his memory, so fraught had it been with profound emotion.

### Nursing in Military Hospitals.

### I.

Strangers entering the wards of a military hospital are usually struck with their bare appearance, due partly to all beds not slept in during the day being rolled up, whilst those occupied have the bedclothes tucked in all round, so that a clear view under the beds can be obtained. In nearly all military hospitals the floors are scrubbed boards; tables, forms, and chairs being of the plainest pattern. Even the armchairs, which really are comfortable and have clean covers once a week, look cold and inhospitable compared with the cosy-looking, bright-coloured seats in ordinary civil hospitals. But with all the bareness of appearance the home feeling is often stronger in a military hospital than in the wards of a general one—sister, orderlies, and patients all being in a corner of their home, the army, knowing and understanding each other's ways, as cannot be the case elsewhere. Wards in a military hospital are of two kinds—nonsisters' and sisters'. In the former, in hospitals where sisters are employed, these are kept for diseases peculiar to men, and any trivial cases for which there may be no vacant beds in a sister's ward. They are nursed by men of the Medical Staff Corps, who also do the whole of the nursing in the smaller military hospitals where sisters are not employed.

The sisters' wards are for general and acute cases, and in them are as large a variety as would be found in any general hospital. Pneumonia, typhoid, fever, rheumatism, tuberculosis, ague, dysentery, abscess of liver, empyema, pleurisy, ophthalmic cases, tonsilitis, accidents, and surgical cases of all kinds, and even chronic spinal cases, paralysis, heart disease, and cancer may be seen here. For though a soldier, unfit for further service, is invalided, yet if not able for moving he is kept many months until arrangements can be made for him, or even until his death. One patient was in for over three years with spinal caries and psoas abscess. Experimental surgery is not allowed in the army, therefore the brilliant operations and splendid cures of civil practice are unknown in them, but all necessary surgery is done in military hospitals. Thus a man may be so injured that it becomes necessary for a limb to be removed, or for some other operation to take place at once. This will be done; but if in a chronic disease, which unfits a man for service, an operation attended with considerable risk to life might alleviate or cure, the patient will probably be invalided and placed (or allowed to place himself) in other hands.

A sister is in full charge of the nursing in her ward, and with tact and determination can take exactly the same position as in a civil hospital. Her duties are to a certain extent ill-defined by regulations, those of surgeon, sister, wardmaster, and orderly overlapping each other to such an extent that without a certain amount of "give and take" spirit, friction arises. But if there is a hearty desire for the comfort of all, she invariably finds her efforts met more than half way. Each sister works, doubtless, according to the traditions of her training school, and it depends upon herself what position she takes. She may make her ward a bright spot in the memory of many a lad, sick and far from home, and by her example help orderlies of the Medical Staff Corps to realise that nursing is a noble profession, not only for women, but also for men, who with brotherly gentleness care for their sick comrades, putting patients first and self last.

For Dress and Uniforms, Notes from St. Helena, In a Lying-in Hospital, &c., see page lii, et seq.

### Dress and Uniforms.

By a Matron and Superintendent of Nurses.

### An Ideal Bag.

It would be difficult, from the point of view of a private or a district nurse, to find anything more tempting than the new bag just brought out by Messrs. Hockin, Wilson, and Co., of 13, New Inn Yard, Tottenham Court Road. The "Acme," as it is called—and it well deserves the name—is of a convenient size, light and portable, and contains every requisite for midwifery and general nursing. All the arrangements are most complete, exciting at once both the curiosity and the admiration of the beholder. Among the contents may be enumerated a two-quart douche, an enema syringe, bath and clinical thermometers, catheters, a kidney bowl, a medicine glass in case, and a breast exhauster, all of which are beautifully mounted and finished off. Receptacles are provided for lint, bandages, and cotton wool, while a sufficient number of stoppered bottles contain the necessary drugs, &c. The bag is morocco-lined, of excellent quality, with a waterproof outside pocket. The cost is comparatively small, which has the great advantage of placing it within the reach of many. For those who prefer to select their own fittings, the same bag can be had empty. The "Midwifery" case is another specialité of Messrs. Hockin and Wilson, and is on similar lines to those prescribed in Germany by the State for the use of midwives. When the lid is raised the sides let down, thus revealing at a glance the whole of the contents. It is fitted with everything necessary for the lying-in-room, and will be found invaluable to those who have devoted themselves to this particular branch of nursing. The case is provided with straps and a handle, which render its transit from place to place an easy matter. Like the bag already described it is cheap, and is quite one of the most reasonable articles of the kind we have had the pleasure of seeing.

### Novelties for Invalids.

A very delightful assortment of fancy and other wools can now be had on application to the Providence Mills Spinning Company. Invalids or nurses, both of whom are proverbially fond of knitting and crotcheting, will find it worth their while to send for samples, as the saving on direct purchase is enormous. Among the fancy varieties may be mentioned the "Astracan," the "Amazon," the "Bechuana," and the "Nabob," all of which are made in every shade of colour, and are beautifully warm and soft. The price also is considerably less than is usually paid for these qualities of wool. We all know how indispensable it is to have a little light work on hand, and the advantages of being able to take up something for a few spare moments without its suffering any detrimental results through being laid down again. On this account, probably, knitting is the favourite occupation of those who are liable to frequent interruption. To such we would strongly recommend a charming heather mixture, which can be had in a variety of blends, and will be found unrivalled in durability and appearance both for socks and stockings. The favourite "Lady Betty" is offered in all shades, to say nothing of the well-known ice, and Pyreneen wools that are in such request for shawls and mufflers. For rugs and couvre-pieds the "Providence" and the "Giant" are most suitable. They are soft and fleecy, and are produced in several exquisite shades; especially worthy of admiration are the olive-greens and the peacock-blues, which would blend harmoniously at the discretion of the worker. We cannot conclude without one word in praise of the crystal wool, which, however, from the fact of its being composed chiefly of silk, is rather more costly. The woollen and cashmere hose supplied by this firm are excellent value for the money, and we would invite our readers to send without delay for samples to the Providence Spinning Mills Company, D Department, Bradford.

### Specialities in Dress Material.

Of all materials which from time to time have left the loom none have acquired such a widespread popularity as serge. It has the virtue of never looking old-fashioned, it is always simple and in perfectly good taste, and is as much in demand in the palace as it is in the cottage. A plain, tight-fitting, tailor-made costume of black or blue serge is, under most circumstances, one of the smartest things any woman can wear, but then it must be faultlessly made. A good deal, however, depends on the quality and appearance of the material as well, and with this idea in our minds we shall be acting wisely if we send to Charles Price and Firm, of Wellington, Somerset, for patterns of his West of England serges. New varieties for autumn and winter wear are now ready, and samples will be sent post free on application to any address. All serges manufactured by this firm are warranted to be pure wool and a fast dye, and will stand any amount of rough usage. The "Cotswold" and the "Orkney" are excellent selections in a heavy make, while for those who require something fine and light the "Brighton" cannot fail to win approval. They are none of them expensive, and vary in price per yard, double width. "Strong union" serges are a special line deserving attention, and are made in black, navy blue, fawn, and grey, 54 inches wide, at a marvellously small cost. For petticoats, charity bundles, or bazaars nothing could be more suitable. Travelling rugs are another speciality in which this firm deals, and are well worth a trial from those who journey much by land or water.

### Ward Shoes.

The patent ward shoes sold by Messrs. R. and J. Dick (60, Holborn) have acquired and are maintaining a deservedly wide and popular reputation. These shoes have the special advantage of fastening by a strap over the instep, which is cut in such a manner as to afford a very substantial support to the foot. This arrangement will be appreciated by all nurses who have a tendency to flat-foot, and it may be safely predicted that no one, after once taking to such ingeniously-constructed articles, will be likely to lay them aside. The shoe is so well shaped that the foot has ample room to expand without presenting a slovenly appearance. Thus to persons engaged in occupations necessitating continuous standing or walking will be found invaluable. The soles are made entirely of leather, and the insoles carefully inserted, so that no ridges or irregularities can press in any way on the foot. Messrs. Dick supply these shoes in two qualities and prices; they likewise guarantee them to wear twelve months.

### Notes from St. Helena.

By Rose A. Blennerhassett, Lady Superintendent of the Civil Hospital.

(*Continued from page* xxxvii).

### II.—FIRST VISIT TO THE HOSPITAL.

Whilst we were hesitating before the door, a polite official came out. He told us he was acting clerk at the hospital, and offered to show us over it. Following him into a flagged passage, we were conducted up two flights of grimy stairs, and found ourselves on a small landing, divided by a low canvas screen, and lighted by a large window. To the left was a wide, arched, doorless opening, passing through which we found ourselves in a male ward containing eight beds. The floor was dirty, the beds covered with tattered woollen quilts of a large tartan pattern; the centre table, enveloped in a sheet, was littered with papers and rubbish of all kinds. The prescription cards hanging over the beds were attached by pieces of silver suture wire! There seemed to be seven patients in the ward, white and coloured. The white men were sailors. Two were suffering from dysentery, and one man was drinking medicine out of a bottle. Though the ward was lofty, well built, well lighted, and cool, even on that hot day, yet the atmosphere was poisonous. In silent dismay we followed our guide up two more flights of stairs to the women's landing. There were about six women in the ward we entered, which was even more evil smelling than the one below. Two other wards were empty. By this time it was nearly twelve o'clock, and curious to see what the dinners were like, we asked for the kitchen. This was a good-sized flagged room on the ground floor. A few embers smouldered in the broken grate but there was no sign of dinner.

### Part II.—Chaos.

From the kitchen we went to the medical store-room, a most hopeless looking place. Instruments, once costly, now broken, rusty, stained with blood, drugs, antiseptic (!) dressings, splints with torn and rotting padding still clinging to them, and appliances of all sorts were thrown together in inextricable confusion. The rubbish of years had accumulated in the hospital, and now, in August, 1894, we are only just getting rid of the last of it. In the palmy days of St. Helena, before M. De Lesseps had imagined the Suez Canal, this island was a very important coaling and provisioning station, and the shipping of many nations thronged the harbour of Jamestown. It was in that golden age that the Civil Hospital was created, at a cost of nearly £8,000. It is well and solidly built, but the site is detestable; it is a miracle of inconvenience and waste of space. For instance, the staircase is so constructed that to carry a patient up on a stretcher half the stretcher must be pushed out of the staircase window. There are no sanitary arrangements attached to the wards. At this moment of writing we can hear the hammers of the workmen who are putting up the first sanitary accommodation on the men's landing. There is no operation-room, and every drop of water has hitherto had to be carried to the top of the hospital, up four flights of stairs.

Evidently plenty of work was in store for us, and we felt that we should be very busy for some months at least. With this conviction in our minds we said good-bye to our guide, and set off for Plantation.

### A Week at Plantation.

The road wound up and up, past hill after hill—always bleak and barren—acre after acre covered with prickly pear growth. Here and there a tiny cottage gleaming white amidst the grey rock, or a clump of aloes, broke the monotony of the view. After an ascent of about an hour we approached Plantation, and then the character of the scenery changed. Trees and verdure, well-wooded hills, came into view, and passing in at the gate we drove through luxuriant vegetation up to the house. It is a grey, square-fronted building, backed by wooded heights, and commanding lovely views of the sea; a beautiful, languorous place, made for dreamy indolence, and a little out of harmony with energetic modern notions. The house was built by the East India Company, probably as a recruiting station for Indian officials, and later on it became the residence of the various governors. It is a roomy old place, erected in the days when solid walls and plenty of elbow room were considered necessaries of life. Of course, it is said to be haunted, the phantom coaches of dead and gone revellers rolling noisily up the drive, whilst doors are solemnly opened by unseen hands, pillows plucked from beneath sleeping heads. The ghosts, however, forbore from troubling us, and day after day slipped by in delightful idleness till the old year made its farewell bow, and work began in earnest in 1894.

### Hospitals in Natal.

### THE GOVERNMENT HOSPITAL AT ADDINGTON.

Addington Hospital was founded on its present site at the time of the Zulu war, and it is the only institution of the kind within reach of the sick on the south-east coast. Its position makes it available for the port and borough as well as for the dwellers in the suburbs of Addington. It is therefore obvious that the nursing department must be an important one, and this was realised by the architect when he designed the present edifice. The nurses' rooms open on to a private balcony from which a glorious view can be enjoyed of bush, coast, and the Indian Ocean. The matron, Miss Aitkin, has been appointed somewhat recently, and under her are eight nurses, trained chiefly in the colonies, and also two probationers. The former are said to be fully qualified, and the authorities are willing to increase the number of probationers until they equal that of the nurses. Food and lodging are provided for the probationers during their period of training. The day nurses are on duty for eleven hours, with time allowed out for meals, and the night nurses are in the wards for ten and a-half hours. We have had several inquiries from time to time about training at this hospital, and we have ascertained that beyond board and lodging there are few attractions to induce Englishwomen to try it, and a voyage on speculation would probably lead to disappointment on arrival, as the supply of the raw material in the colony is sufficient. Since the recent expenditure of £15,000 on new buildings, material changes have taken place in Addington General Hospital, and its present is a great improvement on its former condition. We are indebted to the courteous Agent-General for Natal, Mr. Walter Pease, for the foregoing particulars.

### Appointments.

[It is requested that successful candidates will send a copy of their applications and testimonials, with date of election, to The Editor, The Lodge, Porchester Square, W.]

Dorchester County Hospital.—Miss Jessey Hayes has been appointed Matron of this hospital. She was trained at the Birmingham General Hospital, and afterwards Sister of one of the medical wards. For the last seventeen months Miss Hayes has been night superintendent at the Bristol General Hospital. We congratulate her on her promotion, and wish her success in her new work.

### Wants and Workers.

A lady will be obliged by any reader telling her of a suitable home for a labourer's child of seven, in good health; slightly deficient in intellect; requiring firm, kind treatment and instruction. She is not deaf, but almost dumb. A small sum could be contributed weekly by the mother, who is very anxious for something to be done. Reply to "*Nursing*," The Hospital Office, 428, Strand.

### Pupils in a Lying-in Hospital.

By One of Them.

It was from a sense of duty, and with no thought of enjoying the work, that I entered myself as a pupil-midwife at a lying-in hospital. The training was for three months, and the fee a heavy one; but after all a nurse certainly ought to be capable of helping woman in her hour of greatest need. So I got the necessary extra gowns and aprons, and the neat uniform cap, and a fitted midwifery bag for the district work, and started somewhat loth to learn this particular branch of nursing. It was a shock to discover there were no separate bed-rooms, and that, in spite of the big fee charged, I had to share a room with another pupil. The next shock was to discover there were no regular hours off duty, and that one was liable to be called up for night work. With many an inward groan I betook myself to bed. The hospital was situated in a busy thoroughfare, and the noise kept me awake for a long time, but at last I fell asleep. When I woke it was bright day-light, and my companion was just out of bed.

"The bell has rung," she said. "I will go and get my bath first."

"But it's past seven!" I gasped, looking at my watch, and full of memories of the unearthly hours the nurses in general hospitals have to rise.

"Yes; prayers are at quarter to eight, and breakfast at eight." Well, there was a distinctly homely feeling about these sensible hours; but my companion forgot to warn me about a little ceremony that came between prayers and breakfast. Cleanliness came very close on godliness, and after the last "Amen," all the pupils and nurses stood up and held out their hands; the matron walked down the row and carefully examined the hands and nails—giving a word of advice to this one that her nails should be shorter, or telling the other one to cover up the scratch on her thumb. I was not sure which struck me with most admiration—breakfast at the sensible hour of eight, or the nail parade. After breakfast the pupils made their own beds, and entered the wards at nine. The first thing you did on entering the ward was to turn your sleeves up to your elbows and disinfect in 1 in 1,000 perchloride; before you were allowed to enter the labour-room to attend to a patient you had to scrub your hands and nails for five minutes with carbolic soap in hot water, and then stand one full minute with your hands in the mercury bowl. In fact, the poor germ had no chance whatever! He was swept out of this, and washed off that, and fumed out of the other, till I really wonder the Society for the Prevention of Cruelty to Animals did not take up his case and offer him a little protection.

The system of training the pupils was excellent. On first arrival they took what was called a "nursing case"; that is, they were appointed to receive a patient in the receiving-room and change her clothes, bring her up to the labour-room and attend to her throughout the labour. When the child was born they took the child, and thereafter for fourteen days they had complete charge of the mother and child. During this time they worked in a ward where there were several other patients, so that there was much to be seen and learnt; and whenever a labour was on, a bell was rung, and all might go into the labour-room and see all there was to be seen. In this way a hundred or more cases could be seen in the three months; and at this special hospital there was no medical school, so that there were no men about to take the foremost places; the matron and her assistants delivered nearly all the cases, sending for the physician only when such complications arose as needed forceps, chloroform, &c. If the pupil did well with her nursing case she was given charge of a ward with several beds, and she examined each patient for her ward, and was present throughout the delivery and superintended the dressing of the child, &c. She kept her ward for a fortnight and then, perhaps, had charge of another, or perhaps was sent on the district to attend the mothers in their own homes. And here, let me confess, that I found the double bed-room no inconvenience, for as a rule it was only occupied by one. If I happened to spend the night in bed my companion was probably out on the district, and if she were in bed it was ten to one I was on duty in the labour-room. When we were both in together we had real jolly times comparing our experiences and laughing at the Cox and Box way we shared the room. With the usual hours of bed at 10 p.m. and rise at 7 a.m., and two hours given in the afternoon for study (which one might use for sleep), the night work was not hard as a rule, though if all the cases would occur in the early hours for six nights running as they did sometimes, one got very worn out. We grumbled terribly when there was a rush of work and sleep was cut short, and there was no time to open a book; but we grumbled still more when there came a sudden lull in the work and we slept till we were tired, and studied till our eyes gave out, and then had to sit and stitch bonnet-strings.

The mothers were only kept in hospital for fourteen days if they did well, then they and their babies went off home. Before they went they were expected to attend a thanksgiving service, and they could have their children christened if they like. The mothers on the district were expected to attend at the hospital after they were well and return thanks, and say whether they were satisfied with the services they had received.

The pupil, when first put on the district, goes out with the district midwife, and delivers while the eagle eye of the chief is upon her. If she does pretty well after six or seven times she is sent out alone, with strict orders to send back to the hospital at once if she needs help, or if there is anything she does not understand. If the call comes from an out-door case in the day-time the pupil whose turn it is has to be out of the hospital in five minutes; if the call is at night, she is given ten minutes. On a cold winter's night, to be turned from one's warm bed out into the air in ten minutes is trying to those with weak chests. But in winter you do not suffer so terribly from the dirt of your outdoor patients. After long experience in Holborn and Haggerston, and Stepney, and Bethnal Green, and other districts of London, I here give it as my testimony, that for quantity and variety of insect life Hoxton takes the palm. But this is wandering from the point. It is not only the insects, it is the ignorance and interference of neighbours that make district work so difficult. Shall I ever forget the mother who, when I was proudly practising Créde's method, said to her daughter, "Now cough, my dear; cough well." And the daughter not only coughed, but sort of heaved about the bed and shook off my hold, and hæmorrhage came on. Or the old hag who gave one of my two-day-old babies a lump of bacon rind to suck. But they all did well in spite of everything, and what tales we told one another round the supper-table! How the tales used to grow, and the laughter used to grow, and the matron tried in vain to look severe, and ended by contributing a story herself. It was a charming hospital, and the most interesting nursing experience I ever had. I had been loth to enter it, but I was miserable at leaving it.

### Where to Go.

St. George's Vestry Hall, Little Russell Street, Bloomsbury.—On Wednesday, November 28th, Mrs. Henry Fawcett will lecture at 8.30 p.m. on "The Social Progress of Women during the last Hundred Years."

Trained Nurses' Club, 12, Buckingham Street.—Lecture on Tuesday, November 27th, at 3 p.m., "Nursing of Infectious Diseases of Childhood," by Mrs. Rowlands Humphreys. Tickets for single lecture 1s. 6d.

### Minor Appointment.

London Fever Hospital.—Miss Alice Giles has been appointed Night Superintendent of this hospital. For the last two years she held the post of Sister-in-Charge of the typhoid wards at the General Hospital, Birmingham. We wish her every success in her new work.

### The Muses' Looking Glass.

### A PEEP AT COREA.*

LITTLE enough it is that we know of the small country of Corea, much as we hear its name just now. Up to comparatively modern times, indeed, there were no means of acquiring a clear knowledge through internal sources. The people had no intercourse with the outside world, whilst foreigners were debarred from intruding. The habits and customs of the Coreans demanded that no strangers entered among them, and high gates and city walls represented the attitude of the inhabitants towards other nations. If the Corean left other countries unvisited, so he, too, wished his own to be unexplored of foreign eye. Inhospitable and hostile as this policy would appear at the outset, yet it is a fair example of the inconsistency of the Corean nature. For a more courteous nation towards travellers is not to be found anywhere. Once in the country to-day, a foreigner has the best of everything. We cannot understand the contradiction of their natures any more than we can understand their placid expressionless countenance, or the jumbled-up grammarless language of this far Western nation.

The Land of the Morning Calm, they call their home. The chequered history of the country suggests another appellation. Hedged in between China and Japan, Corean history lies in its geography, and it has been from long times past a prey to the countries it divides. It means a great deal, does this particular and little-to-be-envied locality.

The great contrast with our own institutions lies generally in the social life of the Coreans, in the absolute seclusion of the female portion of the community in particular. You never see a lady in Corea. It is the cardinal point, so to speak, of social etiquette in this kingdom, that the ladies of a household are never seen, indeed, neither are they mentioned. It is only after the curfew strikes that they may take their outing. The male population are then supposed to be at home, and keep indoors. When, as it sometimes happens, a lady does assume a certain independence and ventures to pay a call in the daytime, her doing so involves an elaborate ceremonial, a screened chair and a retinue of ladies' maids. As to the chair coolies, even they must never see her face. When she enters her chair the men must retire, as also when they put her down at her destination. It is only at night time that the native ladies are expected to take their walks abroad, and even then so draped and covered that individual recognition is impossible.

Promotion in official rank is effected through a series of personal examinations. These are described as "being held" in the enclosure behind the palace, and to them come candidates from all parts of the country. The competition is in an open field—some under the trees, some under umbrellas, wealthy ones under tents! His Majesty, who is supposed to be the arbiter of the contest, remains during the examination. The following is an example of the questions set on these occasions, "What does the Yih King say is the duty of children at the death of a father?"

Confucianism is the religion of the country throughout, the real worship of the Coreans being before the tombs and tablets of their ancestors. They are not so zealous a people as their neighbours the Chinese. Temples are few and far between, and not so beautiful as in Japan. Buddhism is traceable in parts; but a great spirit of superstition has usurped the place of a more definite religion. The Coreans are great medicine takers. One hears absurd accounts of this natural trait. It is not so much the actual requirements of health which are studied, as the slavery to custom and habit. Mr. Lowell describes in a humorous manner the philosophy of the medicine-regularly-taken theory. "On every seventh day you rest whether you are tired or not; and on all the other days you work whether you are tired or not. So do we take our medicine once in so many moons, because it is well to observe system, to be regular"—an old Corean pertinently explained to him. It is in fact as an heirloom that medicine is treated in this country. Painted upon suitable spots along the front of the apothecary's shop, Mr. Lowell translates for us the inscription of a legend—"This is the profession left behind by Sin Nong," of whose actual personality, alas! we feel strangely ignorant over here. Surgery, on the other hand, is more advanced in Corea than in China, but it must not approach royalty. No royal person may submit to an operation at the hands of a subject—the etiquette of rank steps in in all its most strained relations between the sovereign and his people.

* "Corea of To-Day." Illustrated. Price 6d. (Nelson and Sons, 1894.)

### A Lecture on Nursing.

IN Miss Honnor Morten's Sunday lecture at the South Place Institute she gave an outline of nursing in the days when the Hindoos and Budhists built hospitals. A quotation from Xenophon of a husband instructing his wife to include the care of her sick slaves amongst her duties had an interest of its own, and then Miss Morten passed on to nursing by the religious orders. In the middle ages the dissolution of the "religious houses" was followed by the disappearance of nursing as a charity, and in the middle ages attendants on the sick were apt to be considered "hired persons, void of commiseration." The progress of nursing at the Seamen's Hospital, Greenwich, a development of the work of the old "Dreadnought," was referred to by Miss Morten, who acknowledged the courtesy of the secretary, Mr. Michelli, in furnishing her with details. Passing from former to present days, the current earnings of nurses in hospital and private work received due attention, and so did the encouragement to thrift, now offered by the Royal National Pension Fund. The training of mental nurses, and the improvements needed in this important work were mentioned in connection with the unusual facilities offered at Berrywood, Northampton, where paying probationers are received.

### An Italian Nurse Murdered.

A REPORT comes from Rome of a cowardly attack made upon a nurse by an ex-patient in the Hospital Santo Spirito. Although the sick in the ward screamed, and nurses attempted to help their companion, no adequate protection was forthcoming, and the murderer was allowed to escape, after stabbing his victim to death. The man, a native of South Italy, had been a very unruly patient, and as soon as his health permitted he was discharged by the doctor. He thereupon vowed vengeance against the nurse (Sister Agostina), whom he blamed for his dismissal, and entering the hospital amidst a crowd of visitors committed this ghastly crime.

### Another Work Society.

THERE is an excellent society at Bolton which has just handed over two hundred garments to the infirmary. The staff highly appreciate this supplement to their own care of the sick, and a very interesting exhibition of the articles sent has been held at the infirmary.

### Notes and Queries.

### Queries.

(44) *No Name.*—Is it necessary for name and address to accompany queries?—*Questioner.*

(45) *Queen's Nurses.*—How can I find out whether any district nursing association is affiliated with the Jubilee Institute?—*Ignoramus.*

### Answers.

(44) *No Name (Questioner).*—Yes, certainly; and we wish that all correspondents would keep this in mind.

(45) *Queen's Nurses (Ignoramus).*—By applying to the inspector, St. Katharine's Hospital, Regent's Park, London,

## Figures and Tables

**Figure f1:**